# The Score

**Published:** 1917-05

**Authors:** Victoria A. H. Duggan


### The Score.

Success does not come by a leap and a bound;
It is simple progression, round upon round.
Each day marks a base, in the big field of Fame;
Each night calls the score, for end of that game.
Each sun spurs us on, with greater endeavor,
To do or to die, in spite of the weather.

If wrongs have been righted and kind acts done,
They score for the game, at each set of sun.
If striving we reach not the coveted goal,
We set ourselves upon this old knoll.
Remember the effort will count just the same,
When all scores are counted at the end of the game.

—*Victoria A. H. Duggan.*

